# Suppressed Electrolyte Decomposition Behavior to Improve Cycling Performance of LiCoO_2_ under 4.6 V through the Regulation of Interfacial Adsorption Forces

**DOI:** 10.1002/advs.202309657

**Published:** 2024-04-23

**Authors:** Chao Sun, Bing Zhao, Zhuan‐fang Jing, Hao Zhang, Qing Wen, He‐zhang Chen, Xia‐hui Zhang, Jun‐chao Zheng

**Affiliations:** ^1^ School of Metallurgy and Environment Central South University Changsha Hunan 410083 China; ^2^ Engineering Research Center of the Ministry of Education for Advanced Battery Materials Central South University Changsha 410083 China; ^3^ National Energy Metal Resources and New Materials Key Laboratory Central South University Changsha 410083 China; ^4^ Key Laboratory of Comprehensive and Highly Efficient Utilization of Salt Lake Resources Qinghai Institute of Salt Lakes Chinese Academy of Sciences Xining 810008 China; ^5^ School of Materials Science and Engineering Central South University Changsha Hunan 410083 China; ^6^ School of Chemistry and Chemical Engineering Hunan University of Science and Technology Xiangtan Hunan 411201 China

**Keywords:** cathode, electrolyte decomposition, interface adsorption, LiCoO_2_, lithium‐ion battery

## Abstract

Alleviating the decomposition of the electrolyte is of great significance to improving the cycle stability of cathodes, especially for LiCoO_2_ (LCO), its volumetric energy density can be effectively promoted by increasing the charge cutoff voltage to 4.6 V, thereby supporting the large‐scale application of clean energy. However, the rapid decomposition of the electrolyte under 4.6 V conditions not only loses the transport carrier for lithium ion, but also produces HF and insulators that destroy the interface of LCO and increase impedance. In this work, the decomposition of electrolyte is effectively suppressed by changing the adsorption force between LCO interface and EC. Density functional theory illustrates the LCO coated with lower electronegativity elements has a weaker adsorption force with the electrolyte, the adsorption energy between LCO@Mg and EC (0.49 eV) is weaker than that of LCO@Ti (0.73 eV). Meanwhile, based on the results of time of flight secondary ion mass spectrometry, conductivity‐atomic force microscopy, in situ differential electrochemical mass spectrometry, soft X‐ray absorption spectroscopy, and nuclear magnetic resonance, as the adsorption force increases, the electrolyte decomposes more seriously. This work provides a new perspective on the interaction between electrolyte and the interface of cathode and further improves the understanding of electrolyte decomposition.

## Graphical abstract

The schematic reveals that the method of coating the LiCoO_2_ (LCO) interface with different electronegative elements (Mg, Sc, Al, Co, Zr, and Ti) can effectively regulate the adsorption force between LCO and the organic components ethylene carbonate (EC). The weak adsorption force can inhibit the decomposition behavior of the electrolyte effectively, and ultimately achieve long‐term cycle stability under high voltage.

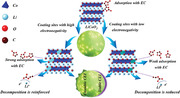

## 1 Introduction

The use of clean energy to fight against environmental degradation has become an irreversible trend in the world. Therefore, it is necessary to rapidly develop advanced energy storage system compatible with clean energy.^[^ ^1^ ^]^ The development of rechargeable lithium‐ion batteries (LIBs) with long‐term cycling life is considered to be the first choice for the next generation of key energy storage devices.^[^ ^2^ ^]^ As a key component of LIBs, LiCoO_2_(LCO) has been commercialized for 30 years, which still occupies the mainstream 3C market with its higher volumetric energy density induced by excellent compaction density than LiNi*_x_* Co*_y_* Mn_1‐_ *_x_*_‐_ *_y_* O_2_ (NCM), LiFePO_4_ (LFP), Li_2_MnO_3_, and Li‐Rich materials.^[^ ^3^ ^]^ Meanwhile, it can achieve more Li^+^ de‐intercalation due to layered structure by increasing the charge cut‐off voltage from 4.2 V (Li, 50%) to 4.6 V (Li, 80%), thereby boosting the energy density of the system to 700 Wh L^−1^ to support the development of clean energy.^[^ ^4^ ^]^

However, it is hard to maintain the stability of long‐term cycle performance of LCO under the voltage condition of 4.6 V because of a series of side reactions triggered by the decomposition of the electrolyte such as the corrosion of HF acid destroys the interface structure to promote the dissolution of the active substance of Co^4+^/Co^3+^, strengthen the nucleophilic attack reaction of interfacial O^2−^, and the oxidation behavior of Co^4+^ and O_2_ to EC.^[^ ^4^, ^5^ ^]^ Meanwhile, the production of insulating substances increases the transport resistance of Li^+^ at the interface.^[^ ^6^ ^]^ In addition, the electrolyte can also gain electrons during the charging‐discharging process, which will reduce the efficiency of the system. Although the irreversible phase transition (O3 to H1‐H3 phase) can cause the capacity fading during cycling,^[^ ^7^ ^]^ the effect is weaker than that of the interfacial reaction on cycling performance.^[^ ^8^ ^]^ Furthermore, the most harmful phase transitions such as layered to disordered rock‐salt phase first occur at material interfaces.^[^ ^4b^ ^]^ Therefore, a large amount of works are currently focused on the breakthrough of the interface bottleneck to achieve long‐cycle stability of LCO at high voltage conditions.

By coating transition metal cations (Al, Nb, Ni–Mn),^[^ ^9^ ^]^ non‐metal (P),^[^ ^10^ ^]^ polymeric materials (polypyrrole),^[^ ^11^ ^]^ ultrathin layer (Li_4_Ti_5_O_12_),^[^ ^12^ ^]^ and fast ion conductors (LATP, LSTP, LiAlO_2_) on LCO interface,^[^ ^2^, ^13^ ^]^ the side reactions at the interface can be effectively blocked, thereby improving the cycle stability of the LCO under high‐voltage. However, these studies only focused on the interfacial stability of cathode materials, but ignored the interaction mechanism between the interface and the electrolyte leading to its degradation. And only a few works have reported the study of related mechanisms, such as the effect of nickel‐rich cathode surfaces on electrolyte decomposition,^[^ ^14^ ^]^ the catalytic effect of LCO interface on the electrolyte in our previous work,^[^ ^6^ ^]^ nucleophilic attack behavior of O_2_^−^ on electrolyte,^[^ ^5b^ ^]^ and dehydrogenation behavior of carbonate solvents.^[^ ^15^ ^]^ Therefore, a further in‐depth understanding of the interaction mechanism between the cathode interface and the electrolyte is of great significance for building an electrolyte decomposition model in an all‐round way and guiding the design of the material structure.

This work directionally modulates the adsorption force between the LCO@X interface coated with different electronegative elements (X = Mg^2+^, Sc^3+^, Al^3+^, Co^3+^, Zr^4+^, Ti^4+^) and the electrolyte. The weak adsorption force can alleviate the excessive decomposition of the electrolyte effectively, and then the cycling stability has been improved. And these elements are selected because they are often used to optimize the electrochemical properties of LCO. DFT shows that as the electronegativity of the coating elements increases, the adsorption force between LCO@X and EC gradually increases, the order of adsorption energy is LCO@Mg (−0.489 eV) < LCO@Sc (−0.503 eV) < LCO@Al (−0.539 eV) < LCO (−0.555 eV) < LCO@Zr (−0.663 eV) < LCO@Ti (−0.730 eV). Meanwhile, time of flight secondary ion mass spectrometry (TOF‐SIMS) further illustrates that the organic layer at the LCO interface would become thinner with the weakening of the adsorption force, the thickness of organic layer at the LCO@Mg (2–3 nm) interface is thinner than LCO@Ti (10 nm). Moreover, the electrochemical performance verifies the trend that the thinner the CEI layer is, the better the cycle stability is. However, LCO@Al defies this trend and exhibits the better cycle stability than LCO@Sc. The reason for this behavior is that LCO@Al can not only alleviate the decomposition of electrolyte, but also effectively suppress the irreversible phase transition. But the effect of electrolyte decomposition on capacity fading is still greater than that of irreversible phase transition, which is illustrated by LCO@Mg with largest irreversible displacement (0.72°) and LCO@Ti with smallest irreversible displacement (0.45°), and LCO@Mg also displays the best capacity retention of 64.1% among them (LCO@X).

## 2 Results and Discussion

**Figure 1:**
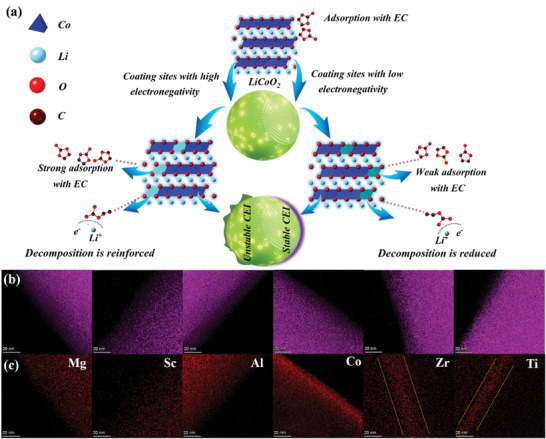
a) Schematic diagram of the effect of different electronegative elements on decomposition degree of EC. b,c) HADDF mode mappings O (purple), Mg, Sc, Al, Co, Zr, and Ti (red) of LCO@Mg, LCO@Sc, LCO@Al, LCO@Co, LCO@Zr, and LCO@Ti cathode.

The degree of disintegration of EC caused by different electronegative elements at the interface of LCO is shown in **Figure** **1**a, and the SEM image and XRD data of LCO are displayed in Figure S1 (Supporting Information). In order to realize the different electronegativity in LCO interface, Mg, Sc, Al, Co, Zr, and Ti elements with different electronegativity can be combined on the LCO interface by means of coating accompanied by 3% content, respectively. These elements are also the most commonly used coating elements currently for optimizing the performance of LCO. And their order of electronegativity is Mg^2+^<Sc^3+^<Al^3+^<Co^3+^<Zr^4+^<Ti^4+^ (Figure S2a, (Supporting Information)).^[^ ^16^ ^]^ After coating with elements on the surface of LCO (note as LCO@X), the morphology and particle size of LCO@X do not change significantly, which is displayed in Figure S2b (Supporting Information). Meanwhile, the influence of different elements on the LCO@X bulk structure is also detected through refined XRD data in Figure S3 and Table S1 (Supporting Information). The results show that coating with foreign elements can change the c‐axis obviously, and then affect the interlayer distance. As the electronegativity of interface coating elements increases, the length of c‐axis is lower. Compared with LCO@Mg (14.0597 Å), the c‐axis of LCO@Sc, LCO@Al, LCO@Co, LCO@Zr, and LCO@Ti are 14.0585 Å, 14.0578 Å, 14.0575 Å, 14.0575 Å, and 14.0480 Å, respectively. This may be attributed to the fact that as the electronegativity of the element increases, its ability to bond with oxygen gradually increases, which in turn gradually changes the original molecular orbital and structure,^[^ ^17^ ^]^ and finally reflects the change of the c‐axis. Furthermore, to visualize the distribution of different elements at the surface of LCO@X, high angle annular dark field (HAADF) mapping is employed (Figure S4, Supporting Information). Meanwhile, Figure 1b shows that the O element (purple) can present a uniform distribution at each LCO@X interface. Figure 1c illustrates that the coating elements of Mg, Sc, Al, Co can be evenly distributed on the interface of LCO@X, but Zr and Ti are enriched at the interface, especially for Ti, which is consistent with previous conclusions.^[^ ^18^ ^]^ In addition, to investigate the transformation effect of elements on the LCO@X interface structure, HRTEM is also used in Figure S5 (Supporting Information). The layered structure of *R*‐3*m* can only be observed in LCO@Co due to the plane of 003 appeared in the two selected regions from fast Fourier transform (FFT) patterns. The reason why the spinel phase is not observed may be that the trace amount of Co_3_O_4_ coating layer after reacting with residual lithium has little impact on the interface structure of the LCO, and it still maintains a layered structure. Other heterogeneous structures have been observed in LCO@X except LCO@Co. LCO@Mg and LCO@Sc exhibit 003 plane with hexagonal *R*‐3*m* structure and 121 plane with spinel phase, respectively. LCO@Al also displays layered structure and two plane of spinel phase with 121 and 110 plane. With the increase of interface electronegativity, the rock salt phase is noticed in both of LCO@Zr and LCO@Ti. The results show that as the electronegativity of the interface elements increases, the original layered structure at the interface of LCO@X is easier to transform into a disordered phase, which may also be attributed to the fact that the molecular orbital and bond length of LCO@X are changed to a greater extent with the increase of the electronegativity of the coating elements.

**Figure 2:**
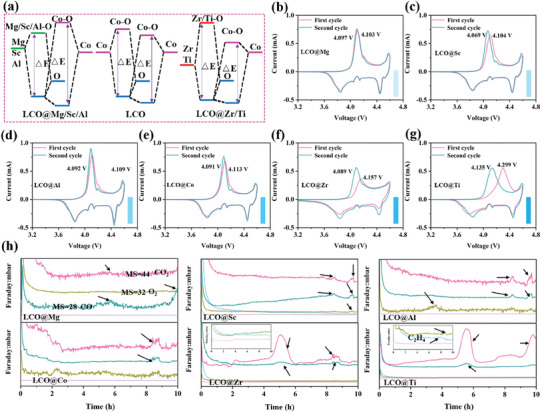
a) Schematics of molecular orbitals of Co‐O‐X (X = Mg, Sc, Al, Co, Zr, and Ti). b–g) Cyclic voltammetry performance for LCO@Mg, LCO@Sc, LCO@Al, LCO@Co, LCO@Zr, and LCO@Ti under the scan rate of 0.1 mV s^−1^ with a voltage range of 3.0−4.6 V for the first two cycles. h) In situ monitoring of gas types and concentrations for the cell system with LCO@Mg, LCO@Sc, LCO@Al, LCO@Co, LCO@Zr, and LCO@Ti cathode.

The schematic diagram of the influence of coating elements on the molecular orbital of LCO@X is shown in **Figure** **2**a. Coating substituents of Zr and Ti, which have greater electronegativity than Co, can snatch electron from Co^3+^ and lower the energy of antibonding states. On the contrary, Mg, Sc, and Al with lower electronegativity than Co, which can donate electron to Co^3+^ and increase the energy of antibonding states.^[^ ^19^ ^]^ Meanwhile, the lowered and raised energy of antibonding states can shift potential positively and negatively, respectively. This is confirmed by cyclic voltammetry tests.^[^ ^20^ ^]^ As shown in Figure 2e, the oxidation peak potential of Co^3+^ is 4.113 V in the first cycle. LCO@Mg, LCO@Sc, and LCO@Al shows the oxidation peak potential are 4.103 V, 4.104 V, and 4.109 V due to the electronegativity of Mg^2+^, Sc^3+^, Al^3+^ is less than Co^3+^ in Figure 2b–d. In contrast, the displayed oxidation peak potential of LCO@Zr and LCO@Ti are 4.157 V and 4.299 V due to the electronegativity of Zr^4+^ and Ti^4+^ are higher than Co^3+^ in Figure 2f,g, and as the increase of electronegativity, the oxidation potential of LCO@X is also more obvious. However, the positions of the oxidation peaks obviously shifted to the left in varying degrees while the cyclic voltammetry test go to the second cycle, especially for LCO@Zr and LCO@Ti. The migration distances of both are 0.068 and 0.164 V, respectively. Since the structure of LCO has almost no attenuation when the initial cycle is performed at low scanning speed, the shift of the peak position may be caused by the decomposition of the electrolyte and the generation of cathode electrolyte interphase (CEI) film.^[^ ^4a^ ^]^ Therefore, in situ differential electrochemical mass spectrometry (DEMS) is tested to illustrate the decomposition behavior of electrolytes in Figure 2h, only a trace amount of CO and CO_2_ are observed in the system with LCO@Mg as the cathode. In the system of LCO@Sc and LCO@Al, the content of CO and CO_2_ gradually increased, and traces of O_2_ is also observed in LCO@Al, which means that the oxidative decomposition degree of the electrolyte gradually increases. With the further strengthening of the interface electronegativity (LCO@Co), the peak signals reflecting the decomposition products CO, CO_2_, and O_2_ of the electrolyte are significantly stronger than that of LCO@Al. Furthermore, as the electronegativity increases to higher level, the peak intensity of CO_2_ is significantly boosted in both of LCO@Zr and LCO@Ti, and the peak of C_2_H_4_ is also observed in LCO@Ti. The interaction electronegativity of LCO@X is closely related to the degree of electrolyte decomposition. The gas generation behavior indicates that with the increase of the interface electronegativity, the decomposition of the electrolyte will be enhanced.

**Figure 3:**
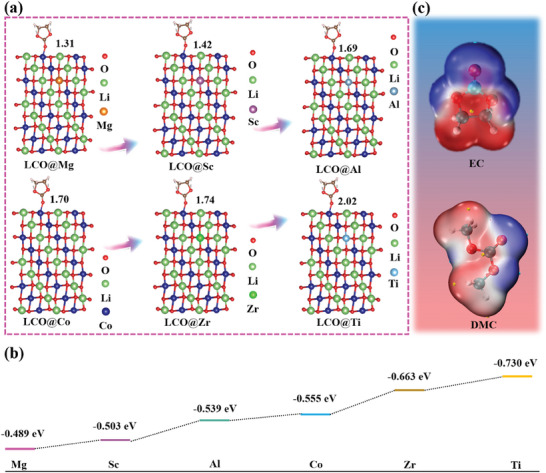
a) The model of calculation adsorption energy between LCO interface with different electronegativities (Mg, Sc, Al, Co, Zr, Ti) and EC. b) Calculation of adsorption energy between LCO@X and EC. c) Prediction of nucleophilic attack sites (blue).

In order to reveal the mechanism of interaction between the intensity of electronegativity and the decomposition behavior of the electrolyte. Density functional theory (DFT) calculates the interaction force between the LCO@X with different electronegativity and EC. Owing to the important reasons for the decomposition of electrolyte are the EC ring‐opening behavior caused by the nucleophilic attack of O^2−^ on EC and the oxidation reaction of EC by O_2_.^[^ ^5^, ^14^ ^]^ We believe that the intensity of the nucleophilic attack and oxidation reaction depends largely on the interaction force between LCO and EC, and a strong adsorption energy may trigger severe ring‐opening and oxidation of EC. The model of calculation adsorption energy between LCO interface with different electronegativities (Mg, Sc, Al, Co, Zr, Ti) and EC is shown in **Figure** **3**a. Figure 3b shows the order of adsorption force between LCO@X and EC is −0.489 eV (LCO@Mg) < −0.503 eV (LCO@Sc) < −0.539 eV (LCO@Al) < −0.555 eV (LCO@Co) < −0.663 eV (LCO@Zr) < −0.730 eV (LCO@Ti). Its calculation formula is as follows:

(1) $$E_{\mathrm{adsorption}}=E_{\mathrm{surface}+\mathrm{EC}}-E_{\mathrm{surface}}-E_{\mathrm{EC}}$$

The results show that with the increase of the electronegativity of the coating elements, the adsorption energy between the interface of LCO@X and EC is gradually enhanced, the higher adsorption capacity may encourage more EC to wrap around the interface, thus enhancing the intensity of nucleophilic attack and oxidation. Ultimately, the decomposition of electrolyte is accelerated and the electrochemical performance is deteriorated. In addition to the adsorption energy, the nucleophilic attack sites of the main solvent molecules of EC and dimethyl carbonate (DMC) in the electrolyte are also investigated. As shown in Figure 3c, the blue regions represent sites where nucleophilic reactions are prone to occur. Compared with DMC, EC is more prone to decomposition and triggers the generation of organic by‐products and gas because of more reaction sites, such as CO, CO_2_, and C_2_H_4_,^[^ ^14^, ^21^ ^]^ thus deteriorating the cycling performance of the LCO at high voltage.

**Figure 4:**
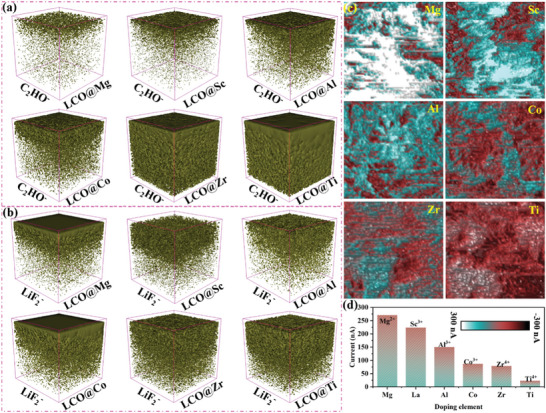
a,b) TOF‐SIMS analysis of the content of C_2_HO^−^ and LiF_2_^−^ fragments at the LCO@Mg, LCO@Sc, LCO@Al, LCO@Co, LCO@Zr, and LCO@Ti interface after 200 cycles at 1C. c) Current distribution at the interface of LCO@Mg, LCO@Sc, LCO@Al, LCO@Co, LCO@Zr, and LCO@Ti after 200 cycles, respectively. d) Comparison chart of current intensity.

The high‐voltage long‐term cycling performance of the LCO@X at 4.6 V with 1 C is further tested to explain the relationship between the interfacial adsorption strength and the electrolyte decomposition behavior. When the cell is cycled for 200 cycles, it is disassembled in the glove box to obtain the positive electrode. After drying, the thickness of the organic component and the conductivity at the interface are tested using TOP‐SIMS and atomic force microscope with conductive mode (c‐AFM), respectively. **Figure** **4**a demonstrates that the thicknesses of the organic composition of C_2_HO^−^ is 1–2, 3–4, 5–7, 6–8, 10, and 10 nm at the interface of LCO@Mg, LCO@Sc, LCO@Al, LCO@Co, LCO@Zr, and LCO@Ti, respectively. The results further verified that the stronger interfacial adsorption energy will lead to more severe electrolyte decomposition behavior. Meanwhile, the fragment of LiF_2_^−^ exhibits the same trend as C_2_HO^−^ on the surface of LCO@X. Figure 4b shows the thicknesses of LiF_2_^−^ at the interface are 2–3 nm (LCO@Mg), 3–4 nm (LCO@Sc), 3–4 nm (LCO@Al), 5–7 nm (LCO@Co), 5–9 nm (LCO@Zr), and 5–9 nm (LCO@Ti) respectively. This may be attributed to the enhancement of the adsorption force of the LCO@X interface, and more EC molecules are likely to entrain the LiPF_6_ being adsorbed to the interface during the charge‐discharge process, thereby accelerating the catalytic decomposition behavior of LiPF_6_ to LiF and HF at the interface of LCO@X,^[^ ^5^, ^22^ ^]^ which has been reported by our previous research.^[^ ^6^ ^]^ Furthermore, HF can corrode the LCO@X matrix so that the interface is damaged, which is illustrated with fragment of CoO_2_^−^ in Figure S6 (Supporting Information). The interfacial damage with a thickness of 1–2 nm for LCO@Mg, LCO@Sc, and LCO@Al, respectively. LCO@Co exhibits a damage thickness of 3–4 nm, and the damaged thickness associated with LCO@Zr and LCO@Ti is 4–6 nm. Moreover, soft X‐ray absorption spectroscopy (sXAS) is adopted to investigate the dissolution degree of Co with total electron yield (TEY) mode after 50 cycles. As shown in Figure S7 (Supporting Information), there is only one peak appearing in LCO@Mg, LCO@Sc, and LCO@Al at 530.5 eV, and it can be attributed to Co^3+^ (eg)–O 2p hybridization.^[^ ^23^ ^]^ Another peak appears in 529.6 eV, which can be attributed to Co^4+^ (eg)–O 2p hybridization and dissolved Co, and as the electronegativity of the interface deepens, the intensity of this peak gradually increases.^[^ ^13a^ ^]^ This also indicates that excessive decomposition of the electrolyte will lead to rapid loss of interfacial active Co. In addition, the influence of the by‐products after electrolyte decomposition on interfacial conductivity is also tested by c‐AFM with SCM‐PIT‐V2 probe. Figure 4c shows the interfacial current intensity after 200 cycles are 270.8 nA (LCO@Mg), 223.6 nA (LCO@Sc), 150.3 nA (LCO@Al), 86.9 nA (LCO@Co), 78.8 nA (LCO@Zr), and 23.1 nA (LCO@Ti), respectively. The gradual decrease of the interfacial current is due to the enrichment of the by‐products with poor conductivity such as C_2_HO^−^ and LiF_2_^−^ produced by the decomposition of the electrolyte at the interface during the charge‐discharge process. This is consistent with the results of TOF‐SIMS. Figure S8 (Supporting Information) also shows that the specific capacities of LCO@X after 200 cycles are 126.1, 127.0, 116.6, 76.6, 42.4, and 36.6 mAh g^−1^, and the corresponding rate of capacity retention are 64.1%, 60.2%, 64.0%, 39.2%, 20.4%, and 17.5% respectively (Figure S8c, Supporting Information). There is a fluctuation in the data during the long‐term cycling process, especially for LCO@Co, LCO@Zr, and LCO@Ti, which may be caused by temperature instability in the test environment or polarization behavior triggered by the cathode and anode plates not fully matching. The result shows that the capacity retention of LCO@X decreases with the enhancement of adsorption capacity between LCO@X and EC, and maintains consistency with the strength of adsorption. However, the capacity retention rate of LCO@Al (64.0%) is acting abnormally, and it should be lower than that of LCO@Sc (60.2%). In order to further investigate the reasons for high‐capacity retention of LCO@Al. Ex situ XRD is tested to analyze the change of bulk structure during charge and discharge process. As shown in Figure S9 (Supporting Information), the phase structure of the LCO@X before and after charging has been detected at 1 C, LCO@X reveals that the offsets of 003 peak are 0.72° (LCO@Mg), 0.56° (LCO@Sc), 0.48° (LCO@Al), 0.78° (LCO@Co), 0.56° (LCO@Zr), and 0.45° (LCO@Ti), respectively. The results illustrate that the elements of Al and Ti has the most obvious inhibitory effect on the structural phase transition, and the phase transition degree is only 0.48° (Al) and 0.45° (Ti).^[^ ^7d^ ^]^ Therefore, LCO@X cycling stability depends not only on the interface stability, but also on the degree of phase transition. However, the interfacial properties induced by electrolyte decomposition remain contribute more to the cycling stability than the structural phase transition, and inhibition of irreversible phase transitions can only have a compensating effect on the cycling stability, which is explained by capacity retention of LCO@Ti and DQ/DV results of LCO@X, respectively. Although LCO@Ti has the smallest phase transition range (0.45°), its capacity retention (0.04%) is the worst in cycling among LCO@X. More, the examination of D*Q*/D*V* derived from the first and 100th charge–discharge curves at 1 C are further presented to illustrate the effect of phase structure on cycling stability in the Figures S10 and S11 (Supporting Information), respectively. The peak of I/I’ represents a conversion of first‐order metal to insulator, and the value of △*V*_I‐I’_ represents the influence of the irreversible phase transition induced by the high voltage condition exceeding 4.5 V on the structural decay,^[^ ^7^, ^24^ ^]^ and higher values symbolize faster degradation of the structure. Figure S10 (Supporting Information) demonstrates that the value of △*V*_I‐I’_ are 0.0851 V (LCO@Mg), 0.0806 V (LCO@Sc), 0.0718 V (LCO@Al), 0.0879 V (LCO@Co), 0.0733 V (LCO@Zr), and 0.0724 V (LCO@Ti) in the first curves. LCO@Al still demonstrate mastery over irreversible phase transitions, and the conclusion is consistent with ex‐situ XRD data. However, the ability to control the deformation of the phase structure of LCO@Al is clearly weakened as described in Figure S11 (Supporting Information) after 100th cycles. The value of △*V*_I‐I’_ are 0.1156 V (LCO@Mg), 0.1214 V (LCO@Sc), 0.1316 V (LCO@Al), 0.1660 V (LCO@Co), 0.1691 V (LCO@Zr), and 0.1920 V (LCO@Ti), respectively. The result is consistent with the decomposition degree of electrolyte in Figure 4. This illustrates that although the inhibition of the irreversible phase transition can improve the stability at the beginning of the cycling, the contribution of the interface properties to the stability is more significant as the number of cycles increases, which can be ascribed to electrolyte decomposition products enriched at the interface may break the protective barrier of Al to the bulk structure. Moreover, Figure S8a (Supporting Information) also illustrates that the capacity fading rate per revolution of LCO@Al in 100–200 cycles (0.473 mAh g^−1^) is significantly larger than that in 1–100 cycles (0.156 mAh g^−1^).

**Figure 5:**
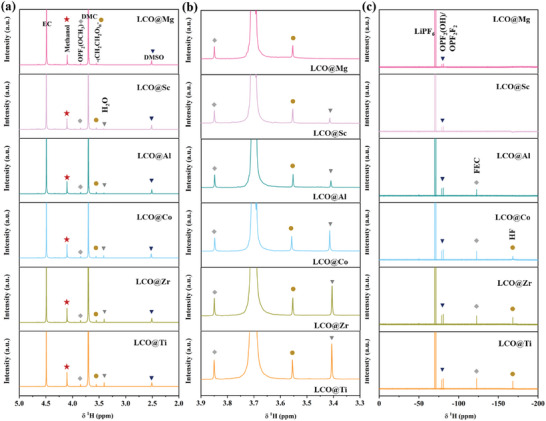
a) ^1^H NMR signal of electrolyte extracted from the cell of LCO@Mg, LCO@Sc, LCO@Al, LCO@Co, LCO@Zr, and LCO@Ti to DMSO‐d_6_ solvent. b) The amplified ^1^H NMR signal of electrolyte from Figure 5a. c) ^19^F NMR signal of electrolyte extracted from the cell of LCO@Mg, LCO@Sc, LCO@Al, LCO@Co, LCO@Zr, and LCO@Ti to DMSO‐d_6_ solvent.

To further visualize the decomposition behavior of the electrolyte and to ascertain in detail the components of the electrolyte decomposition products during cycling. Liquid nuclear magnetic resonance (NMR) is tested during the charging–discharging process with 0.2 C (3.0–4.6 V) after 50 cycles in the **Figure** **5**. In Figure 5a, the DMSO‐d_6_ solvent captured decomposition products from glass fiber separator to form solution after 50 cycles is analyzed because of long‐term cycling (200 cycles) can obscure subtle differences, which can capture small molecule fragments keenly. The ^1^H NMR spectrum of LCO@X shows the signals of EC (4.49 ppm), methanol (4.10 ppm), OPF_2_(OCH_3_) (3.85 ppm), DMC (3.70 ppm), ─(CH_2_CH_2_O)*_n_* ^−^ (3.55 ppm), and DMSO (2.51 ppm) in Figure 5a,^[^ ^25^ ^]^ respectively. And the signal of 2.51 can be attributed to impurities of DMSO in DMSO‐d_6_ solvent.^[^ ^26^ ^]^ The electrolyte decomposition behavior can also be reflected by the changes in the signal intensity of methanol (4.10 ppm) and H_2_O (3.40 ppm). With the intensification of the electrolyte pulverized, the peaks of methanol and H_2_O gradually strengthens (LCO@Ti > LCO@Zr > LCO@Co > LCO@Al > LCO@Sc > LCO@Mg). Besides, the signal of ─(CH_2_CH_2_O)*_n_* ^−^ also shows the same trend in the magnified graph (Figure 5b). This result explains that the decomposition of the electrolyte would lead to the accumulation of harmful substances, which would further accelerate the decomposition of the electrolyte and cause a vicious circle. The H_2_O, which caused by the oxidation of EC,^[^ ^5^, ^14^ ^]^ would trigger a sequential reaction as its content increases, such as the following:

(2) $$\mathrm{LiP}F_{6}\to \mathrm{LiF}+PF_{5}$$

(3) $$PF_{5}+H_{2}O\to 2\mathrm{HF}+\mathrm{OP}F_{3}$$

(4) $$\mathrm{OP}F_{3}+H_{2}O\to \mathrm{HF}+\mathrm{OP}F_{2}\left(\mathrm{OH}\right)$$

This is probably the reason why the peak of OPF_2_(OH) gradually increases in the Figure 5c.^[^ ^27^ ^]^ Meanwhile, this would also trigger the generation of HF to destroy the interface of LCO@X and lead to the formation of insulating species,^[^ ^28^ ^]^ such as LiF, which is one of the factors that lead to poor cycling performance at high voltage. In addition, fluoroethylene carbonate (FEC) is also observed in the Figure 5c, which can be attributed to the reaction between vinylene carbonate (VC) and HF.^[^ ^14^ ^]^

**Figure 6:**
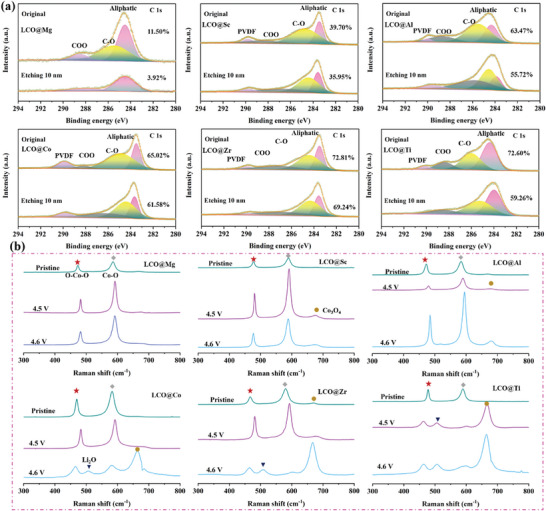
a) XPS sputtering spectra of C 1s for the surface of LCO@Mg, LCO@Sc, LCO@Al, LCO@Co, LCO@Zr, and LCO@Ti after 200 cycles. b) Ex situ Raman measurement for LCO@Mg, LCO@Sc, LCO@Al, LCO@Co, LCO@Zr, and LCO@Ti at pristine (3.0 V), 4.5 V, and 4.6 V after 50 cycles.

In order to be able to quantitatively describe the content of by‐products that caused by the decomposition of electrolyte at the interface of LCO@X, sputtering X‐ray photoelectron spectroscopy (XPS) and ex situ Raman are further tested in **Figure** **6**. As shown in Figure 6a, before etching, C 1s spectra illustrates the carbon content is 11.50% (LCO@Mg), 39.70% (LCO@Sc), 63.47% (LCO@Al), 65.02% (LCO@Co), 72.81% (LCO@Zr), 72.60% (LCO@Ti) at the interface of LCO@X. The C content generated by the decomposition of electrolyte induced by strong adsorption (LCO@Zr/LCO@Ti) is 6 times than that of weak adsorption (LCO@Mg). This is very detrimental to electrochemical stability. Meanwhile, the peaks at 283.48, 284.38, 287.38, and 289.93 eV are assigned to aliphatic, C─O, COO, and PVDF, respectively.^[^ ^29^ ^]^ And the signals of aliphatic, C─O, and COO can be attributed to the breakdown of EC and DMC organic solvent. After sputtering at 10 nm, the carbon content covering the interface of LCO@X is reduced to 3.92% (LCO@Mg), 35.95% (LCO@Sc), 55.72% (LCO@Al), 61.56% (LCO@Co), 69.24% (LCO@Zr), and 59.26% (LCO@Ti) in Figure 6a. COO and C─O almost disappear at the interface of LCO@Mg. However, both of them can still exist very clearly at the interface of LCO@Ti after sputtering 10 nm. This indicates that the organic molecules produced by the excessive decomposition of the electrolyte can be stubbornly enriched at the interface of LCO@X, which is consistent with the result of TOP‐SIMS. In addition, F 1s spectra is also shown to explain the change of F content before and after sputtering in Figure S12 (Supporting Information). Only the peak of LiF (684.7 eV) is observed in the interface of LCO@Mg before and after sputtering.^[^ ^30^ ^]^ In addition to LCO@Mg, the peak of Li*_x_* F*_y_* PO*_z_* (686.7 eV) can still be observed after sputtering the interface of other cathode materials by using the Ar etching method. Figure S12 (Supporting Information) expresses the F content of the interface before etching is 25.58% (LCO@Mg), 26.58% (LCO@Sc), 33.20% (LCO@Al), 30.99% (LCO@Co), 32.65% (LCO@Zr), and 39.82% (LCO@Ti). After sputtering 10 nm, the F content on the interface is 17.91% (LCO@Mg), 25.80% (LCO@Sc), 23.82% (LCO@Al), 27.21% (LCO@Co), 31.04% (LCO@Zr), and 37.22% (LCO@Ti). This phenomenon is consistent with the variation of C content and also suggests that the decomposition of LiPF_6_ is reinforced along with the enhancement of the decomposition behavior of the organic solvent and their by‐products can exist in the interface of LCO@X compatible with each other. Moreover, ex‐situ Raman testing can further illustrate the violent degree of the boundary reaction for LCO@X with LiPF_6_ solution after 50 cycles in Figure 6b. The mechanism of the reaction is as follows:^[^ ^31^ ^]^

(5) $$3\mathrm{LiCo}O_{2}+Li^{+}+e^{-}\to Co_{3}O_{4}+Li_{2}E$$

In the pristine state, the Raman signal of LCO@X exhibits two peaks at 470.1 and 583.0 cm^−1^, which are assigned to O─Co─O and Co─O in octahedron, respectively. After being charged to 4.5 V, although the positions of the O─Co─O and Co─O barely shifted in LCO@X, the peak of Co_3_O_4_ appeared in LCO@Sc, LCO@Al, LCO@Co, LCO@Zr, and LCO@Ti, and is most evident in LCO@Ti, it means that the interfacial reaction has been occurred at the interface of these cathode when charging to 4.5 V in LiPF_6_ solution. With further deepening of the charging level to 4.6 V, the peak of Co_3_O_4_ is clearly boosted in other materials except LCO@Mg. Not only the signal of Co_3_O_4_, but also the peak of Li_2_O (506.9 cm^−1^) are further observed in LCO@Co, LCO@Zr, and LCO@Ti. For LCO@Ti, the pronounced peak of Co_3_O_4_ is the earliest observed at 4.5 V, and the peak shifts of O─Co─O and Co─O are the greatest during the voltage charging to 4.6 V. This result provides proof that the strong adsorption energy between interface and electrolyte leads to the failure of LCO@X interface. In‐situ electrochemical impedance spectra (EIS) is also tested to comprehensively consider the effects of by‐products from electrolyte decomposition and interface failure on the interface impedance. Figure S13 (Supporting Information) presents the change trend of the interfacial impedance of CEI film (note as R_CEI_) during charging process for LCO@X.^[^ ^7c^ ^]^ Although the R_CEI_ value of LCO@Mg at 3.9 V (376.2 Ω) and 4.0 V (132.3 Ω) is the highest compared to other materials, but after 4.1 V, it drops rapidly to 76.26 Ω and maintains the minimum value during the subsequent charging process from 4.1 to 4.6 V. In contrast, although the *R*_CEI_ value of LCO@Ti is low in the early stage of charging, as the charging depth increases, the *R*_CEI_ value gradually increases. After charging to 4.3 V, the *R*_CEI_ value shows a maximum value compared to others. Meanwhile, the order of *R*_CEI_ is consistent with the degree of electrolyte decomposition after 4.3 V, which indicates that *R*_CEI_ can directly reflect the severity of electrolyte decomposition at voltages higher than 4.3 V. As the discharge proceeds in Figure S14 (Supporting Information), the value of *R*_CEI_ increases gradually and mutations occur at 4.0 V, especially for LCO@Ti. The *R*_CEI_ value of LCO@Ti rapidly increases from 51.66 Ω (4.0 V) to 413.4 Ω (3.8 V), and the increment is 361.74 Ω. And the increment for others is 17.35 Ω (LCO@Mg), 40 Ω (LCO@Sc), 24 Ω (LCO@Al), 126.3 Ω (LCO@Co), 127.4 Ω (LCO@Zr), respectively. LCO@Mg also shows the minimum value. Although value of LCO@Al is smaller than LCO@Sc, the order of *R*_CEI_ value is consistent with the charging process after discharging to 3.8 V. The results further show that the decomposition degree of the electrolyte will directly affect the variation of *R*_CEI_, and the violent interface decomposition behavior can lead to the increase of *R*_CEI_ within a certain voltage range.

## 3 Conclusion

This work focuses on the interaction between the interface of LCO and the electrolyte. By coating different electronegative elements (Mg, Sc, Al, Co, Zr, Ti) on the interface of LCO, the adsorption capacity of the interface and the electrolyte is changed, thereby effectively inhibiting the decomposition of the electrolyte and promoting cycling stability of LCO under high voltage conditions. Using DFT revealed that as the interface electronegativity increases, its adsorption energy with the electrolyte gradually increases. The powerful adsorption force will enhance nucleophilic attack and oxidative reaction behavior, thereby accelerating electrolyte disintegration during the cycling performance, producing a large number of inactive by‐products, which has been verified by TOP‐SIMS, c‐AFM, NMR, sXAS, DEMS, and sputtering XPS. In contrast, the weak adsorption energy between LCO@Mg and the electrolyte caused by elements with low electronegativity can alleviate its excessive decomposition. In addition, the ex‐situ XRD results further illustrate that electrolyte decomposition is more destructive to the performance of LCO than irreversible phase transition. We earnestly hope that this work will stimulate the study of the factors causing electrolyte decomposition and deeply analyze the influence of electrolyte decomposition on interface attenuation.

## 4 Experimental Section

### Preparation of LCO@X (X = Mg, Sc, Co, Al, Zr, Ti)

In this work, the baseline LCO was synthesized by Co_3_O_4_ and Li_2_CO_3_ with the molar ratio of 1:1.5 under 1000 °C for 12 h. For the synthesis of LCO@X, MgO, Sc_2_O_3_, Al_2_O_3_, Co_3_O_4_, ZrO_2_, and TiO_2_ were selected as coating additives respectively. Meanwhile, in order to enhance the efficacy of the elements, the proportion of the elements that need to be added in LCO was controlled at 3 wt%. After MgO (0.102 g), Sc_2_O_3_ (0.095 g), Al_2_O_3_ (0.117 g), Co_3_O_4_ (0.084 g), ZrO_2_ (0.084 g), and TiO_2_ (0.103 g) were uniformly mixed with LCO (2 g) by solid‐phase grinding method. Then, the mixture was placed in a muffle furnace and heated to 900 °C for 10 h, note as LCO@Mg, LCO@Sc, LCO@Al, LCO@Co, LCO@Zr, and LCO@Ti. After the temperature drops from 900 °C to 30 °C, all of them was taken out and crushed to obtain the cathode.

### Physical Characterization

The changes in the morphology and phase structure of different cathode were observed by field‐emission scanning electron microscopy (FE‐SEM, JSM‐7900F, JEOL) and powder X‐ray diffraction with Cu Kα (λ = 1.54 Å) radiation (PANalytical, X'PRO Pert), respectively.

The changes of surface signals in nanoscale for LCO and LCO@X were obtained using High‐resolution transmission electron microscopy (HRTEM, Thermo Fischer Talos F200x). And HAADF mode images of different elements distributed at the LCO interface was also observed with TEM. In order to detect the decomposition behavior of the electrolyte at the material interface and the interface composition information after cycling, X‐ray photoelectron spectroscopy (XPS, ThermoFisher Scientific Escalab 250Xi, USA), Ex‐situ Raman data (DXR, *λ* = 532 nm), time of flight secondary ion mass spectrometry (PHInanoTOF II), atomic force microscope (AFM, dimension ICON) accompanied by conductive mode (c‐AFM) with a SCM‐PIT‐V2 probe, liquid nuclear magnetic resonance (LNMR, AVANCE III 500 M) were employed. Meanwhile, in situ differential electrochemical mass spectrometry (DEMS, Hiden HPR40) also was used in combination with electrochemical workstation (Bio‐Logic, VMP‐300, France) to detect the type and content of gas produced by the decomposition of the electrolyte during the charging–discharging process with a voltage range of 3.0–4.6 V.

### Electrochemical Characterization

The model for electrochemical performance testing was a coin cell (CR2023). Here, the slurry on the surface of Al foil was coated and dried at 60 °C for 12 h as cathode, which contains LCO/LCO@X, conductive carbon (Super p) and polyvinylidene difluoride (PVDF) with a ratio of 8:1:1. The polypropylene membrane as separator with a diameter of 12 mm, and lithium metal as anode accompanied by the thickness of 0.5 mm. Electrolyte contains 1.0 m LiPF_6_ in ethylene carbonate/dimethyl carbonate (volume ratio of EC:DMC = 1:1). All the materials mentioned above were assembled in a glove box with a water content of 0.01 ppm and an oxygen content of 0.01 ppm to obtain coin cell. The electrochemical performance data of rate, galvanostatic charge and discharge, and long‐term cycling were tested on the LAND test system (CT2001A, China). The data of in situ electrochemical impedance spectroscopy (in situ EIS) from 100 kHz to 0.01 Hz, cyclic voltammetry (CV) with a scan rate of 0.1 mV s^−1^, and Linear sweep voltammetry (LSV) were collected by electrochemical workstation (Bio‐Logic, VMP‐300, France) under 3.0–4.6 V.

### Theoretical Calculation

In this work, VASP package was adopted to calculate the energy of adsorption between LCO/LCO@X and EC. the structures of LCO, LCO@X, and ethylene carbonate (EC) were first optimized, and the energy of each atom should be accurate to 10^−4^ eV. After that, the energy of *E*_EC_, *E*_surface_, and *E*_(EC+surface)_ were calculated. Finally, the interfacial adsorption energy between LCO@X and EC were obtained using the formula of *E*_adsorption_ = *E*_(EC+surface)_ − *E*_EC_ − *E*_surface_. In addition to the interfacial adsorption energy, the differential charge density was further delved to explain the evolution behavior at the electronic level. Meanwhile, the ways of projector‐augmented wave (PAW) were employed to describe the interaction mechanism between ions and valence electrons and GGA also used to elaborate generalized functions of exchange correlation with PBE mode. The selection of crystal planes (104) and the determination of atomic number as a period were consistent with our previous research. And 3 × 3 × 1 k‐point mesh was used for Monkhorst‐Pack scheme.

## Conflict of Interest

The authors declare no conflict of interest.

## Supporting information

Supporting Information

## Data Availability

The data that support the findings of this study are available on request from the corresponding author. The data are not publicly available due to privacy or ethical restrictions.
