# Supplementary material for: Suppressed Electrolyte Decomposition Behavior to Improve Cycling Performance of LiCoO2 under 4.6 V through the Regulation of Interfacial Adsorption Forces
Source: Adv Sci (Weinh). 2024 Apr 23;11(25):2309657. doi: 10.1002/advs.202309657 (PMC11220708; doi:10.1002/advs.202309657)
Supplement: Supplementary file 1 — Supporting Information [file ADVS-11-2309657-s001.pdf]

ADVANCED SCIENCE
Open Access

Supporting Information

for *Adv. Sci.*, DOI 10.1002/advs.202309657

Suppressed Electrolyte Decomposition Behavior to Improve Cycling Performance of $LiCoO_2$ under 4.6 V through the Regulation of Interfacial Adsorption Forces

*Chao Sun, Bing Zhao, Zhuan-fang Jing, Hao Zhang, Qing Wen, He-zhang Chen, Xia-hui Zhang and Jun-chao Zheng**

Suppressed Electrolyte Decomposition Behavior to Improve Cycling Performance of $LiCoO_2$ under 4.6 V through the Regulation of Interfacial Adsorption Forces

Chao Sun[1,2,3], Bing Zhao[4], Zhuan-fang Jing[4], Hao Zhang[5], Qing Wen[1,2,3], He-zhang Chen[6], Xia-hui Zhang[1,2,3], Jun-chao Zheng[1,2,3*]

[1].School of Metallurgy and Environment, Central South University, Changsha, Hunan 410083, China

[2].Engineering Research Center of the Ministry of Education for Advanced Battery Materials, Central South University, Changsha 410083, China

[3].National Energy Metal Resources and New Materials Key Laboratory, Central South University, Changsha 410083, China

[4].Key Laboratory of Comprehensive and Highly Efficient Utilization of Salt Lake Resources, Qinghai Institute of Salt Lakes, Chinese Academy of Sciences, Xining 810008, China

[5].School of Materials Science and Engineering, Central South University, Changsha, Hunan 410083, China

[6]. School of Chemistry and Chemical Engineering, Hunan University of Science and Technology, Xiangtan, Hunan 411201, China

Corresponding Authors (E-mail): jczheng@csu.edu.cn (j. c. Zheng)

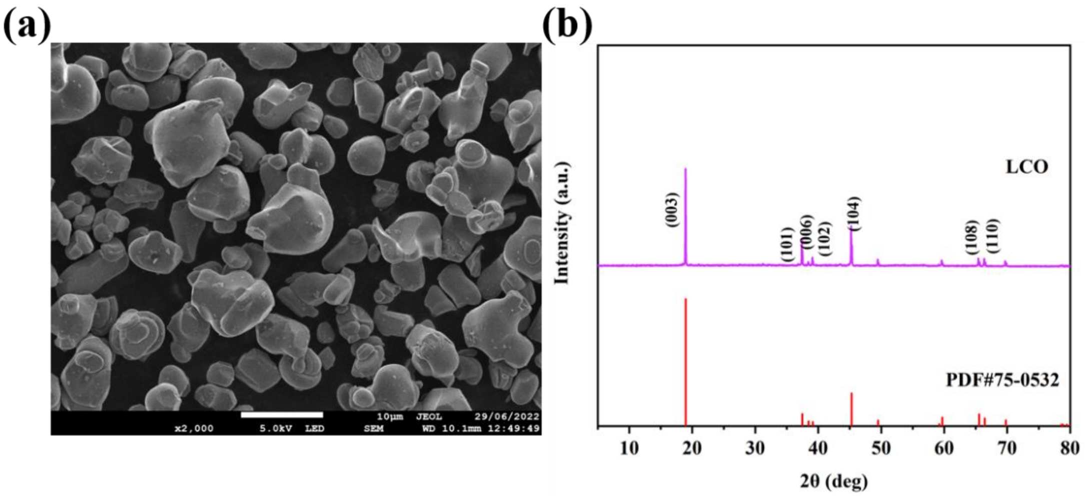

Figure S1. (a) SEM image of LCO. (b) XRD data of LCO.

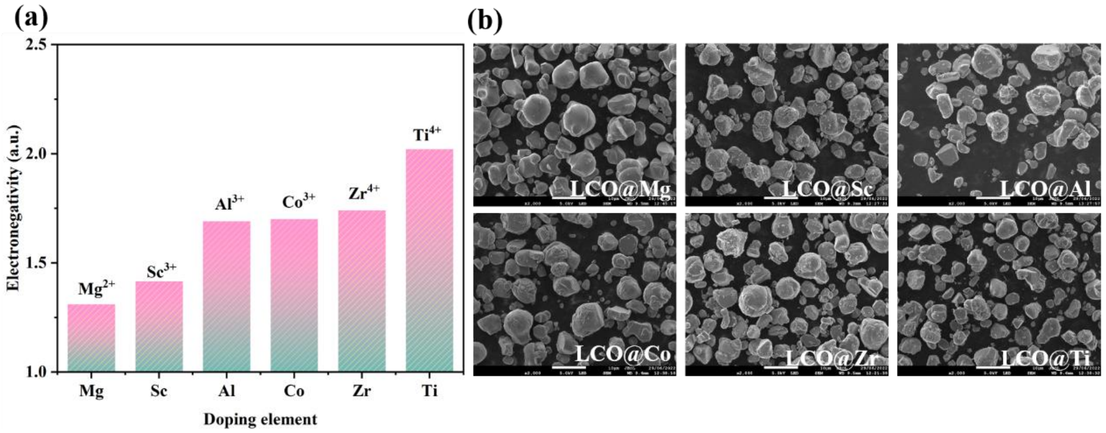

Figure S2. (a) Comparison of electronegativity for Mg^{2+}, Sc^{3+}, Al^{3+}, Co^{3+}, Zr^{4+}, and Ti^{4+}. (b) SEM images of LCO@Mg, LCO@Sc, LCO@Al, LCO@Co, LCO@Zr, and LCO@Ti particles.

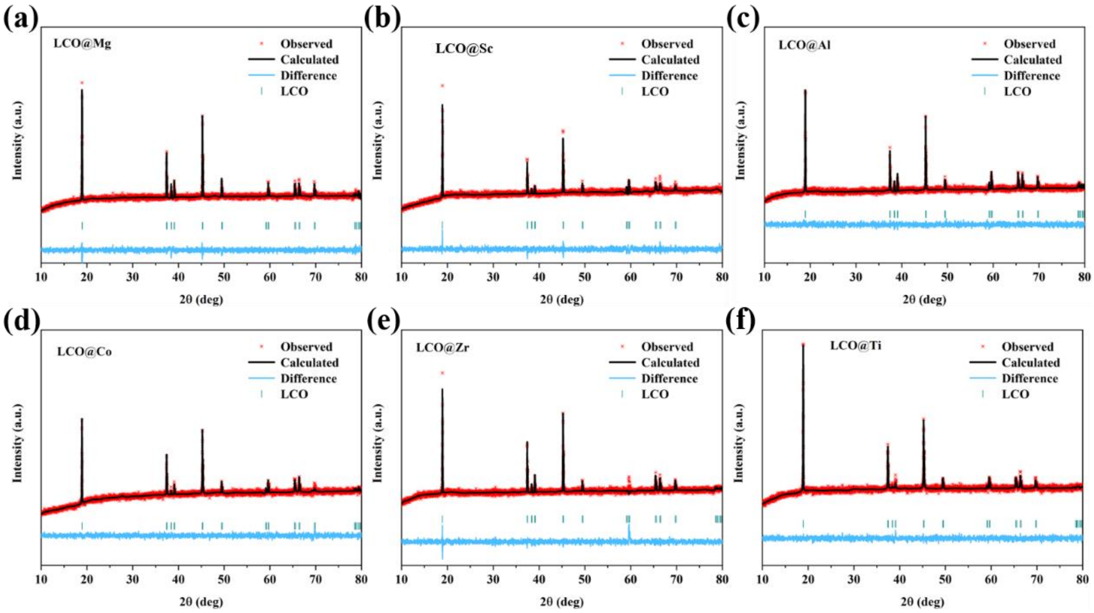

Figure S3. (a-f) Refined XRD from LCO@Mg, LCO@Sc, LCO@Al, LCO@Co, LCO@Zr, and LCO@Ti.

Table S1. Refined XRD data.

Sample	LCO@Mg	LCO@Sc	LCO@Al	LCO@Co	LCO@Zr	LCO@Ti
***a*, *b* [Å]**	2.8146	2.8141	2.8142	2.8146	2.8141	2.8148
***c* [Å]**	14.0597	14.0585	14.0578	14.0575	14.0575	14.0480
**α, β [°]**	90	90	90	90	90	90
**γ [°]**	120	120	120	120	120	120
**R_p [%]**	2.02	2.52	2.04	1.95	2.43	2.69
**R_{wp} [%]**	2.58	3.27	2.59	2.50	3.49	3.58

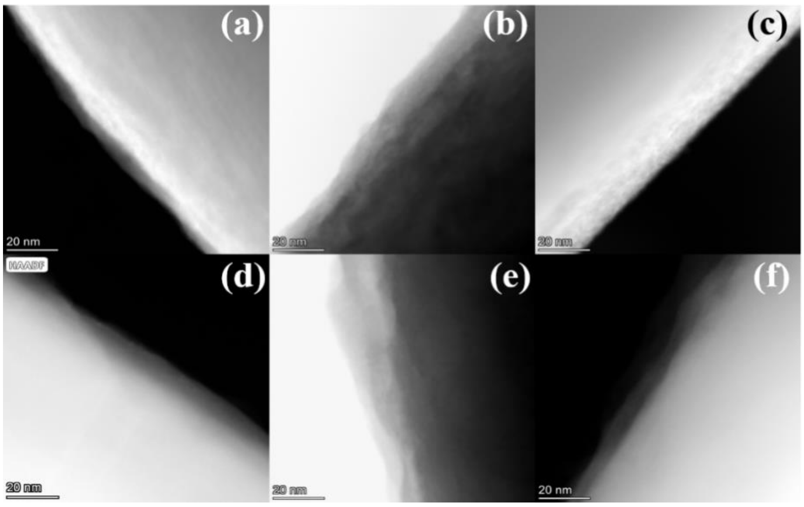

Figure S4. (a-f) TEM images of LCO@Mg, LCO@Sc, LCO@Al, LCO@Co, LCO@Zr, and LCO@Ti.

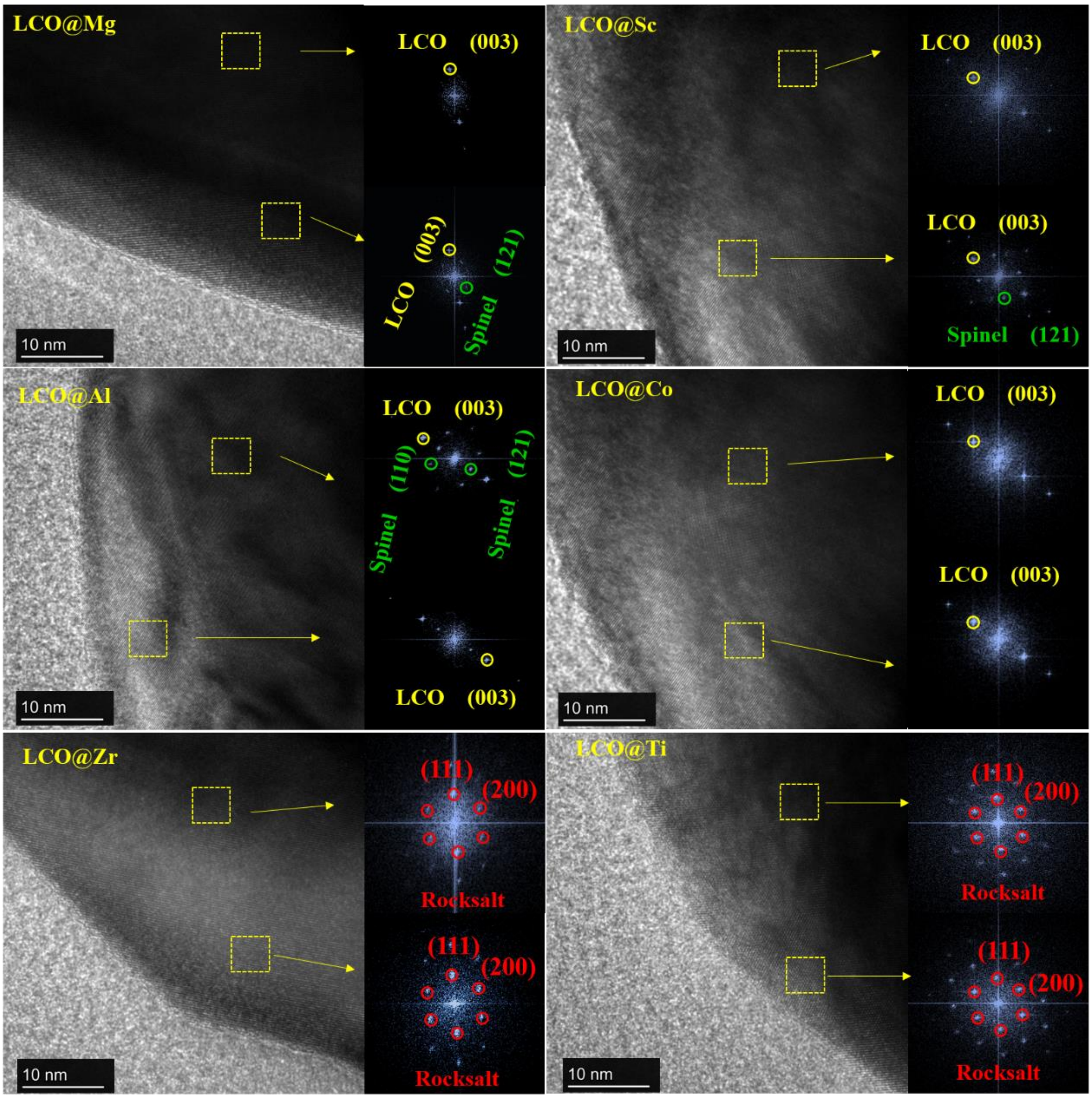

Figure S5. HRTEM images of LCO@Mg, LCO@Sc, LCO@Al, LCO@Co, LCO@Zr, LCO@Ti and the corresponding FFT pattern from selected regions.

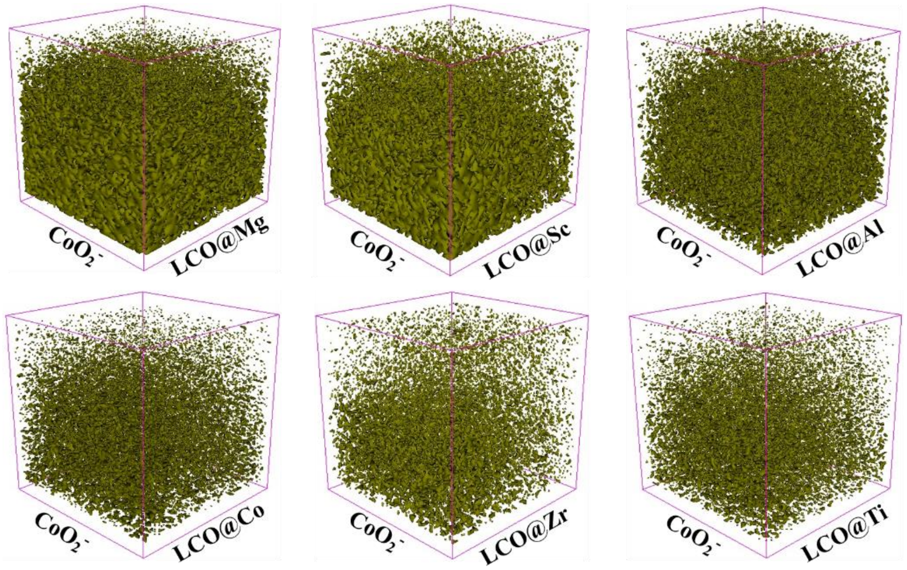

Figure S6. TOF-SIMS analysis of the content of CoO_2^- fragments at the LCO@Mg, LCO@Sc, LCO@Al, LCO@Co, LCO@Zr, and LCO@Ti interface after 200 cycles at 1C.

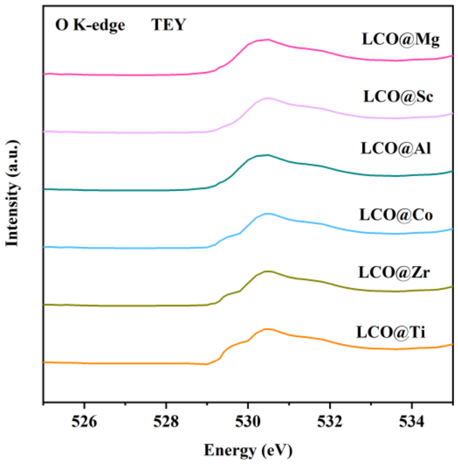

Figure S7. O K-edge XAS spectra of LCO@Mg, LCO@Sc, LCO@Al, LCO@Co, LCO@Zr, and LCO@Ti after 200 cycles.

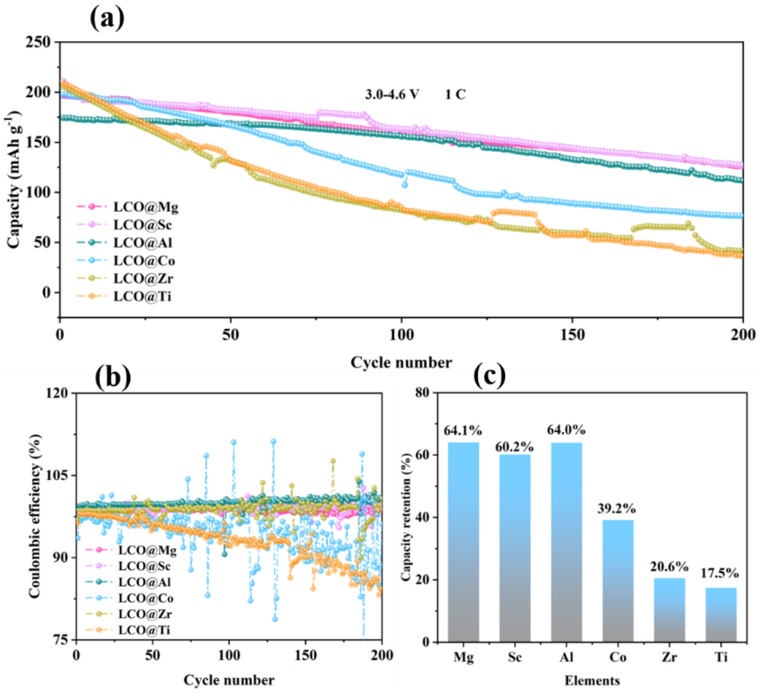

Figure S8. (a) Long-term cycling performance of LCO@Mg, LCO@Sc, LCO@Al, LCO@Co, LCO@Zr, and LCO@Ti with a voltage range of 3.0−4.6 V at 1 C. (b) The corresponding coulombic efficiency during the cycling process. (c) Comparison of the capacity retention of LCO@Mg, LCO@Sc, LCO@Al, LCO@Co, LCO@Zr, and LCO@Ti after 200 cycles.

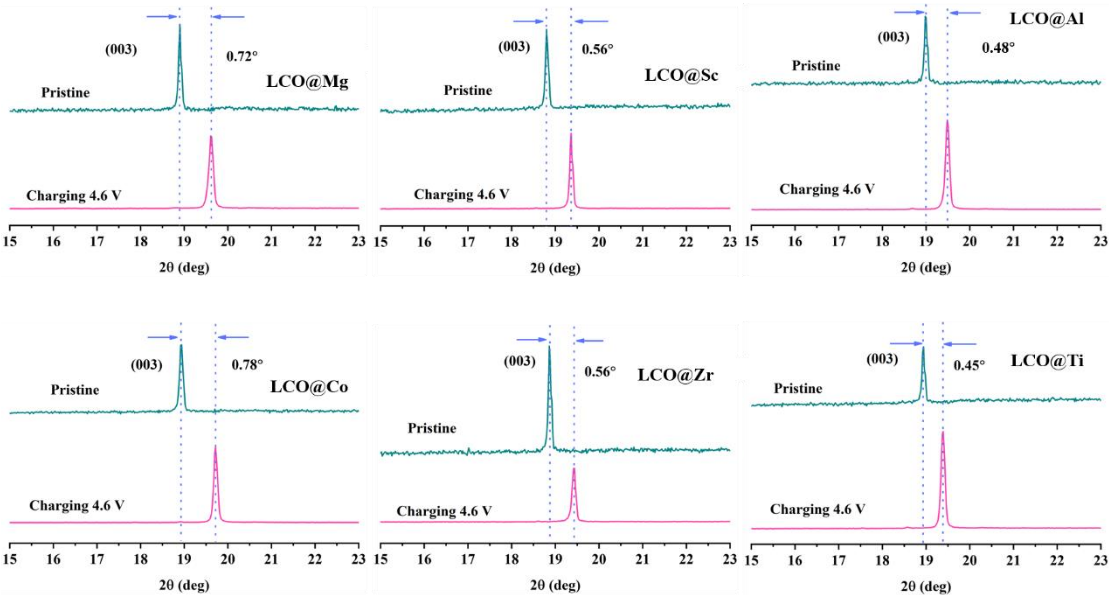

Figure S9. Ex-situ XRD measurement before (3.0 V) and after (4.6 V) charging for LCO@Mg, LCO@Sc, LCO@Al, LCO@Co, LCO@Zr, and LCO@Ti.

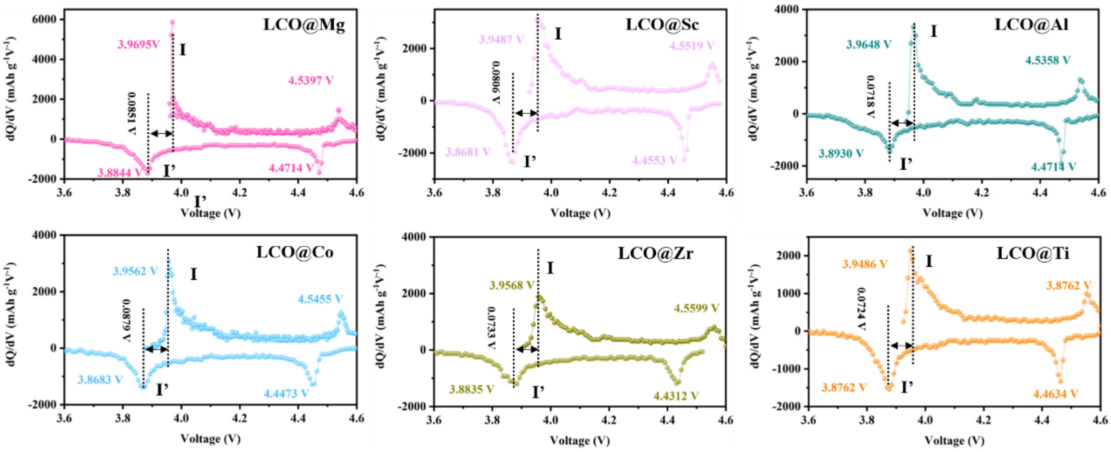

Figure S10. The dQ/dV curves for the first cycle at 1 C in a voltage range of 3.0–4.6 V.

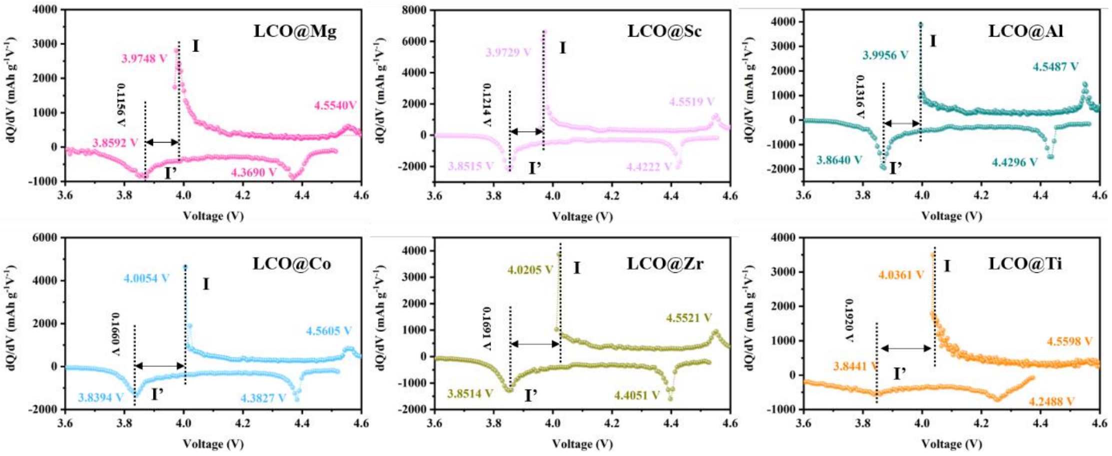

Figure S11. The dQ/dV curves after 200 cycles at 1 C in a voltage range of 3.0–4.6 V.

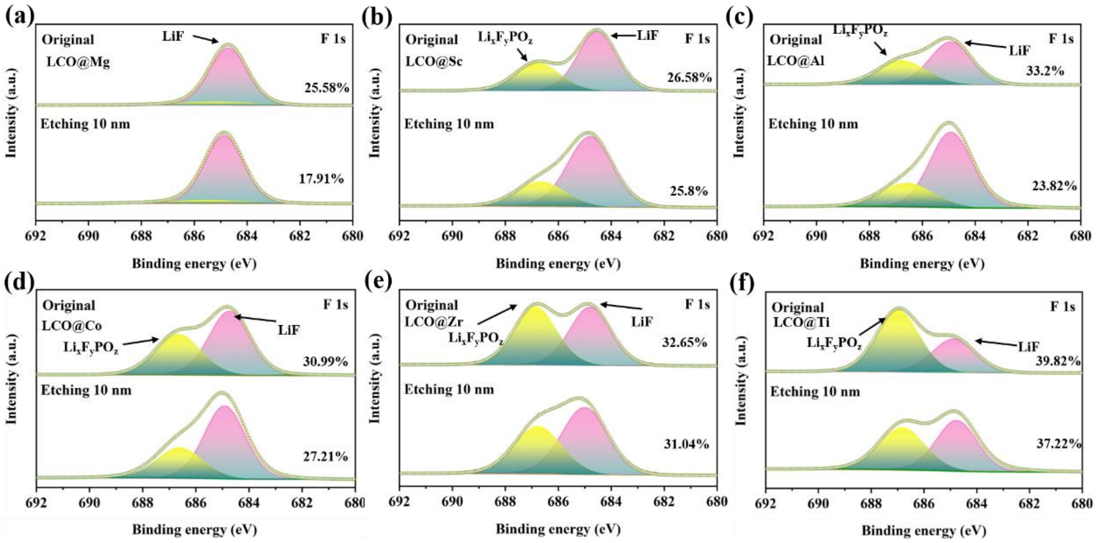

Figure S12. XPS sputtering spectra of F 1s for the surface of LCO@Mg, LCO@Sc, LCO@Al, LCO@Co, LCO@Zr, and LCO@Ti after 200 cycles.

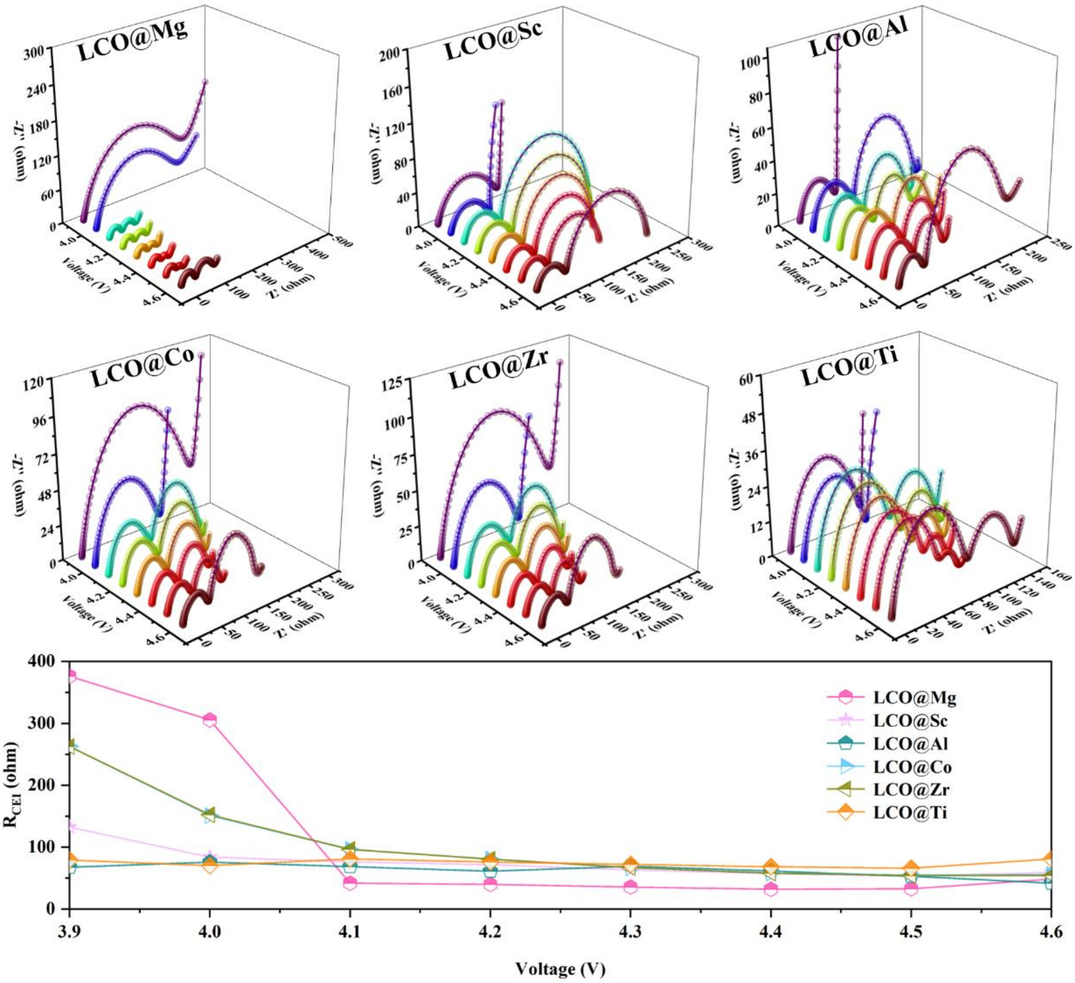

Figure S13. In-situ EIS profiles from 100 kHz to 0.01 Hz during charging process from 3.9 V to 4.6 V for LCO@Mg, LCO@Sc, LCO@Al, LCO@Co, LCO@Zr, LCO@Ti, respectively.

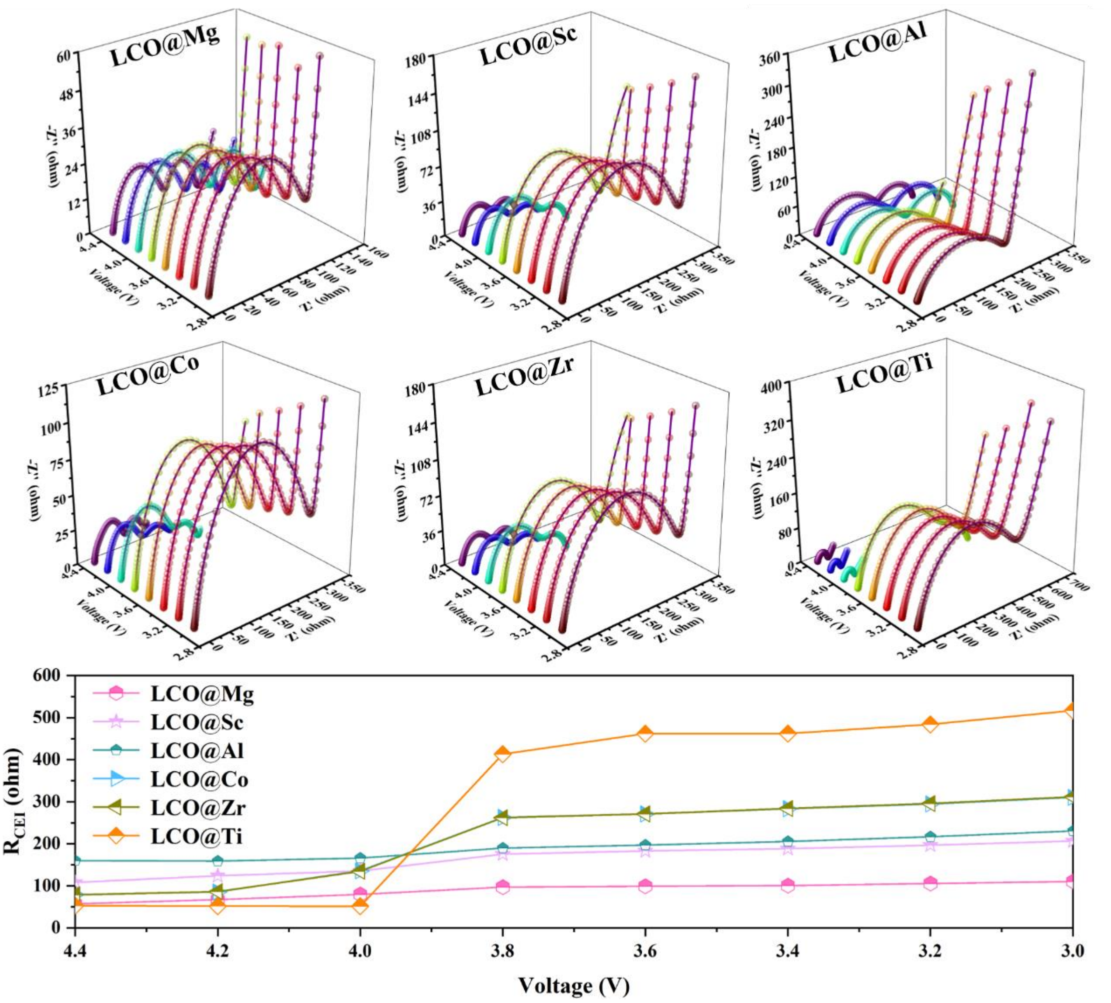

Figure S14. In-situ EIS profiles from 100 kHz to 0.01 Hz during discharging process from 4.4 V to 3.0 V for LCO@Mg, LCO@Sc, LCO@Al, LCO@Co, LCO@Zr, LCO@Ti, respectively.
